# A novel reindeer cyclone optimization algorithm (RCOA)

**DOI:** 10.1038/s41598-025-97069-1

**Published:** 2025-04-11

**Authors:** Gopal Chaudhary, Bharat S. Rawal

**Affiliations:** 1 School of Engineering & Technology, Vivekananda Institute of Professional Studies - Technical Campus, Delhi, India; 2 Grambling State University, Grambling, LA USA

**Keywords:** Engineering, Mathematics and computing

## Abstract

This paper introduces the Reindeer Cyclone Optimization Algorithm (RCOA), a novel metaheuristic optimization technique inspired by the survival behavior of reindeer during predator attacks in formation cyclonic storms. RCOA imitates the defense-centric cooperative behavior of reindeer, where individuals cluster together to withstand external threats. This behavior is analogous to the optimization process where exploration (global search for exploring new areas) and exploitation (local refinement to copy or learn from neighbor in cyclonic form) are carefully balanced. The algorithm has been extensively evaluated against 14 unimodal and multimodal benchmark functions and 4 real-world complex optimization problems. RCOA demonstrates a moderate improvement of around 5–12% over other algorithms such as PSO, DE, COA and GSA on unimodal functions. On multimodal functions, RCOA shows more competitive performance, especially in terms of stability, with an improvement of around 10–15% in accuracy and consistency compared to WDO and PSO. The algorithm is evaluated using the CEC’17 benchmark suite with 50 dimensions and compared against different well-established optimization algorithms, including WOA, PSO, GSA, and DE. Experimental results demonstrate that RCOA outperforms existing methods on multiple test functions by achieving superior convergence speed and solution accuracy. The Wilcoxon Signed-Rank test confirms the statistical significance of RCOA’s performance, indicating its robustness and reliability in handling diverse optimization landscapes. The findings suggest that RCOA is a competitive optimization method suitable for a wide range of real-world applications.

## Introduction

Metaheuristic algorithms have become essential tools for tackling complex optimization problems, especially those that are nonlinear, multimodal, and constrained^1^. Their flexibility, adaptability, and ease of implementation have made them popular across various scientific and engineering fields. Nature-inspired algorithms, such as Genetic Algorithms (GA)^2^, Particle Swarm Optimization (PSO)^3^, Ant Colony Optimization (ACO)^4^, and more recently the Firefly Algorithm (FA)^5^ and the Whale Optimization Algorithm (WOA)^6^, have been successfully applied to a wide range of optimization challenges. However, these methods often struggle to maintain a consistent balance between exploration (the broad search of the solution space) and exploitation (the focused search around promising regions), particularly in high-dimensional or highly multimodal search spaces^7^. This imbalance can lead to premature convergence or inefficient search behavior.

To address these limitations, we introduce the Reindeer Cyclone Optimization Algorithm (RCOA), a novel metaheuristic technique inspired by the survival behavior of reindeer during predator attacks and cyclonic storms. In nature, reindeer herds form tight clusters in response to external threats, leveraging a cooperative defense strategy that parallels the exploration-exploitation dilemma in optimization. This behavior allows the herd to dynamically balance between evading threats (exploration) and maintaining cohesion for survival (exploitation). RCOA adopts this adaptive clustering strategy to achieve a more efficient and robust search process, enhancing both the global search capabilities and the local refinement of solutions.

The mathematical foundation of RCOA is presented in this paper, along with its implementation on 14 unimodal and multimodal benchmark functions and four real-world complex optimization problems. Rigorous comparisons with established algorithms such as PSO, WOA, Differential Evolution (DE)^8^, Gravitational Search Algorithm (GSA)^9^, and other state-of-the-art methods like Grey Wolf Optimizer (GWO)^10^ and show that RCOA consistently delivers competitive performance. Specifically, RCOA demonstrates superior accuracy, convergence stability, and solution quality, particularly in complex, multimodal search spaces.

The results of this study highlight RCOA’s ability to efficiently navigate challenging optimization landscapes, offering a promising alternative for solving real-world engineering problems. By leveraging the cooperative behavior of reindeer, RCOA achieves a dynamic balance between exploration and exploitation, outperforming several traditional and contemporary metaheuristics in both precision and consistency. This positions RCOA as a robust and scalable solution for addressing a wide variety of optimization challenges.

### Contributions of the paper

The novelty of RCOA lies in its biologically inspired search mechanism, which differs from existing metaheuristics in the following ways:

- RCOA is modeled after the natural defense strategy of reindeer herds, where individuals form a swirling motion to protect weaker members, leading to a unique balance between exploration and exploitation.
- Unlike traditional algorithms with fixed exploration-exploitation settings, RCOA adjusts its search dynamics throughout the iterations, allowing for more efficient navigation of complex landscapes.
- Incorporating Lévy flight helps RCOA escape local optima by introducing random, large jumps, improving global search capability.
- The algorithm simulates the swirling movement of reindeer, which refines promising solutions by systematically adjusting positions in a controlled manner.
- Agents update their positions based on interactions with strong candidates rather than just the single best solution, fostering diverse learning and reducing stagnation.
- Instead of blindly following the best candidate, each agent moves in proportion to its distance from high-performing solutions, ensuring a smooth balance between global and local search.

The rest of the paper is structured as follows. Section “Related work” describes the related study. Section “Proposed algorithm: reindeer cyclone optimization algorithm (RCOA)” proposes the Reindeer Cyclone Optimization Algorithm (RCOA) and its inspiration. Section “Mathematical model of RCOA” introduces the Mathematical model of RCOA. Section “Results and discussion” gives the result and discussion section. Section 6 concludes the main findings and suggests directions for future research.

## Related work

Metaheuristic algorithms have gained widespread attention in the optimization community because of their ability to solve complex, non-linear, and multimodal problems in various domains. These algorithms are particularly advantageous because they do not rely on gradient information, making them highly effective in escaping local optima, a significant limitation in traditional optimization methods. This section discusses several well-established nature-inspired algorithms and their advancements, providing a foundation for the development of the Reindeer Cyclone Optimization Algorithm (RCOA).

The earliest and most widely used category of metaheuristic algorithms is evolution-based methods. Genetic Algorithms (GA), introduced by Holland^2,11^, simulate the process of natural selection. GA evolves a population of candidate solutions through selection, crossover, and mutation operators to find the optimal solution. Other evolutionary methods include Evolution Strategy (ES)^12^ and Genetic Programming (GP)^13^, which have demonstrated efficacy in various optimization problems by employing biological evolution principles.

Recently, Differential Evolution (DE)^14^ has gained significant attention due to its simplicity and efficiency. DE is known for its ability to handle continuous optimization problems by relying on differential operators to enhance population diversity. Despite their successes, evolution-based methods often suffer from slow convergence and are prone to premature convergence in complex, multimodal search spaces.

Physics-based algorithms mimic natural physical phenomena and processes to drive the optimization search. Simulated Annealing (SA)^15^ was one of the earliest algorithms in this category, inspired by the annealing process in metallurgy. SA is effective in avoiding local optima by probabilistically accepting worse solutions during the search, which improves the exploration phase.

Another prominent physics-based approach is the Gravitational Search Algorithm (GSA)^16^, where candidate solutions are considered as masses attracting each other based on Newtonian gravity. Over time, the search converges toward more massive objects, corresponding to better solutions. Similarly, the Charged System Search (CSS)^17^ and Central Force Optimization (CFO)^18^ rely on other physical forces to guide optimization.

While these algorithms have provided robust solutions in various applications, they often struggle with balancing exploration and exploitation, especially in high-dimensional search spaces.

Swarm-based algorithms, inspired by the collective intelligence of animals, have become some of the most popular and successful metaheuristics for optimization. Particle Swarm Optimization (PSO)^19^, proposed by Kennedy and Eberhart^3^, mimics the social behavior of bird flocking or fish schooling. PSO excels in exploration by maintaining individual and global best solutions, although it can sometimes converge prematurely in multimodal problems.

Ant Colony Optimization (ACO)^20^, introduced by Dorigo et al., mimics the foraging behavior of ants by using a pheromone-based communication system. ACO has been widely applied to combinatorial optimization problems, especially in routing and scheduling tasks.

Recent swarm-based methods have shown improvements in performance. For example, the Whale Optimization Algorithm (WOA)^21^ simulates the hunting behavior of humpback whales and has been particularly successful in solving multimodal problems. Likewise, the Grey Wolf Optimizer (GWO)^10^ imitates the hierarchical leadership and hunting strategies of wolves. Firefly Algorithm (FA)^22^, inspired by the bioluminescent communication of fireflies, has also demonstrated strong convergence properties in multimodal optimization.

Several novel algorithms have emerged in recent years, pushing the boundaries of metaheuristic performance. Teaching-Learning-Based Optimization (TLBO) and its variants^23,24^ draws on the concept of knowledge transfer between teachers and students to optimize complex problems. Similarly, Imperialist Competitive Algorithm (ICA)^25^ mimics socio-political imperialism to find optimal solutions. Both of these methods have shown competitive performance in benchmark problems, particularly in their ability to balance exploration and exploitation. There are several recent algorithms available in literature which are useful in optimation problems^26–30^.

Another noteworthy recent method is the Moth-Flame Optimization (MFO)^31^, inspired by the navigation method used by moths called transverse orientation. The algorithm has shown superior performance in high-dimensional problems. Similarly, the Salp Swarm Algorithm (SSA)^32^ and Seagull Optimization Algorithm (SOA)^33^ introduce novel swarm behaviors that have demonstrated improvements in convergence speed and accuracy.

The literature also includes several machine learning and deep learning methods for optimization, in addition to metaheuristic approaches^34^. Nature-inspired algorithms have remarkably efficiently solved complex optimization problems, particularly in dynamic and uncertain environments. Recent studies have explored various bio-inspired strategies, such as gecko-inspired locomotion for robotic coordination^35^, cockroach bio-robot navigation for autonomous path planning^36^, and multimaterial soft robotic hand control^37^, showcasing the effectiveness of nature-inspired solutions in engineering applications. Similarly, UAV communications and pursuit-evasion models utilizing swarm intelligence and deep reinforcement learning (DRL)^38,39^ highlight the significance of optimizing collective movements for enhanced performance. In the realm of network optimization, heuristic-based techniques have been used for identifying influential nodes in social networks^40^, mining spatial co-location patterns^41^, and designing hybrid optimization-based adversarial attack strategies^42^, reinforcing the role of intelligent search mechanisms in high-dimensional spaces.

Despite advances in metaheuristics^43^, maintaining a robust balance between exploration and exploitation remains a central challenge, particularly in highly multimodal and complex search spaces. Most of the discussed methods exhibit weaknesses in convergence stability, often leading to suboptimal solutions. Reindeer Cyclone Optimization Algorithm (RCOA) is introduced to address these limitations by leveraging the adaptive survival behavior of reindeer herds during cyclonic storms, which naturally balance global exploration and local exploitation. The cooperative cyclone formation in reindeer herds not only enables effective defense mechanisms but also mirrors the optimization process of dynamically searching through and refining promising regions in the search space.

In comparison to the aforementioned algorithms, RCOA offers a more dynamic and adaptive balance, ensuring both precision and consistency across various optimization challenges. By combining the strengths of swarm-based search and dynamic adaptability, RCOA is poised to outperform many existing algorithms in both accuracy and robustness.

This study positions RCOA as a competitive and promising addition to the growing family of nature-inspired metaheuristic algorithms, with particular strength in solving real-world and multimodal optimization problems.

**Fig. 1:**
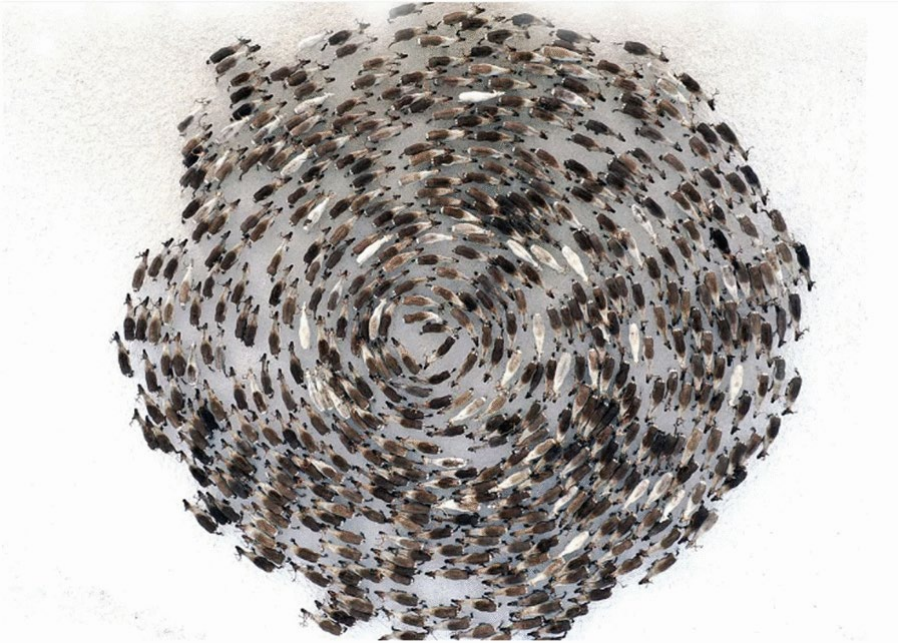
Reindeers forming a cyclone-like structure.

## Proposed algorithm: reindeer cyclone optimization algorithm (RCOA)

### Inspiration

The Reindeer Cyclone Optimization Algorithm (RCOA) is inspired by the unique defense mechanism of reindeer herds during a threat. In nature, reindeers form a cyclone-like structure where the younger and older members of the herd are kept in the center for protection, while the stronger reindeers form an outer circle to defend against predators as shown in Fig. 1. This behavior is analogous to the optimization process where exploration (global search) and exploitation (local refinement) are carefully balanced. The center of the herd corresponds to the optimum region, and the outer reindeers represent the search agents dynamically exploring and converging towards this optimum. This collective motion enhances global search (exploration), while their synchronized movements during foraging improve local search (exploitation). The algorithm models reindeer survival strategies such as exploration, exploitation, and random walk behaviors based on Levy flight.

## Mathematical model of RCOA

The optimization process involves the following steps:

### Position update equation

This step reflects the initial, scattered positions of reindeers before a threat arises. The random positions ensure that the algorithm begins with agents distributed across the search space, allowing a thorough exploration of the solution space. This ensures that the algorithm starts with a wide diversity of possible solutions (like reindeers being spread out in an open field), crucial for avoiding local optima. The position of each agent (reindeer) is updated based on a combination of exploration, exploitation, and sometimes a random walk. Let $X_i^{(t)}$ be the position of the $i$-th agent at iteration $t$ in a $D$-dimensional search space. The new position at iteration $t+1$ is updated as:

1 $$\begin{aligned} X_i^{(t+1)} = X_i^{(t)} + \alpha \cdot r_1 \cdot (X^* - X_i) + \beta \cdot r_2 \cdot (X_i - X_{\text {neighbor}}) + \gamma \cdot D \cdot e^{b t} \cos (2 \pi t) + {\mathcal {L}}(\beta ) \end{aligned}$$

where:

- $X_i^{(t)}$—Current position of the $i$-th agent.
- $X^*$—Best-known position in the current iteration.
- $X_{\text {neighbor}}$—Position of a randomly selected neighboring agent.
- $\alpha$ and $\beta$—Learning rates for exploration and exploitation.
- $$r_1, r_2 \sim U(0,1)$$—Random vectors.
- $D = \Vert X_i - X^*\Vert$—Euclidean distance to the best agent.
- $b$ - Spiral constant, $t \sim U(0,1)$—Random parameter.
- $\gamma$—Controls the influence of spiral movement.
- ${\mathcal {L}}(\beta )$—Lévy flight random step.

### Exploration term

The reindeers in the outer ring constantly move and adjust their positions to protect the herd. In the algorithm, exploration represents the broader search for new potential solutions, mimicking the outer reindeer’s task of scanning the environment for threats. The exploration equation allows agents to explore new areas by pulling the search agents toward the current best solution. As the iteration progresses, the learning rate ($\alpha$) decreases, making exploration more focused on the most promising areas of the search space.

In the exploration phase, each agent moves towards the best-known solution with a learning rate that decreases over time. The exploration term is defined as:

2 $$\begin{aligned} \text {Exploration} = \alpha \cdot r_1 \cdot (X^* - X_i) \end{aligned}$$

where $\alpha$ is a constant influencing the exploration step.

The exploration learning rate $\alpha$ is given by:

$$\begin{aligned} \alpha = \text {lr}_{\min } + (\text {lr}_{\max } - \text {lr}_{\min }) \cdot \left( 1 - \frac{k}{K_{\text {max}}}\right) \end{aligned}$$

Where:

- $\text {lr}_{\min }$ and $\text {lr}_{\max }$ are the minimum and maximum learning rates.
- $k$ is the current iteration.
- $K_{\text {max}}$ is the maximum number of iterations.
- $r_1$ is a random vector of size *D* uniformly distributed in [0, 1].

This equation ensures that the exploration phase dominates at the beginning of the search and gradually decreases over time.

### Exploitation term (neighbor-based)

This is analogous to the inner ring of reindeers clustering more tightly to defend vulnerable members. Here, exploitation is the process of refining the search around promising solutions, focusing on local optimization by intensifying the search within a smaller region. The exploitation strategy is based on neighboring agents. It encourages the search agents to “copy” or learn from their neighbors, leading to convergence around good solutions. As iterations progress, $\beta$ increases, indicating a shift from broad search to intensified local refinement.

In the exploitation phase, agents adjust their positions based on the location of a randomly selected neighboring agent. The exploitation term is defined as:

3 $$\begin{aligned} \text {Exploitation} = \beta \cdot r_2 \cdot (X_i - X_{\text {neighbor}}) + D \cdot e^{b t} \cos (2 \pi t) \end{aligned}$$

The exploitation learning rate $\beta$ increases over time and is defined as:

$$\begin{aligned} \beta = \text {lr}_{\min } + (\text {lr}_{\max } - \text {lr}_{\min }) \cdot \frac{k}{K_{\text {max}}} \end{aligned}$$

- $r_2$ is a random vector of size *D* uniformly distributed in [0, 1].
- ${X}_{\text {neighbor}}$ is the position of a randomly selected neighboring agent.

### Levy flight (random walk)

To balance exploration and exploitation and avoid local optima, RCOA introduces a random walk mechanism based on Lévy flight. With a small probability $p = 0.1$, the agent updates its position as:

4 $$\begin{aligned} x_i^{(t+1)} = x_i^{(t+1)} + \text {L}\acute{\textrm{e}}\text {vy}(\beta ), \end{aligned}$$

where Lévy flight is modeled as:

5 $$\begin{aligned} \text {L}\acute{\textrm{e}}\text {vy}(\beta ) = 0.01 \cdot \frac{u}{|v|^{\frac{1}{\beta }}}, \end{aligned}$$

with:

- $\beta = 1.3$ is the Lévy flight parameter.
- $u \sim N(0, \sigma _u^2)$, where $\sigma _u$ is computed as:
  6 $$\begin{aligned} \sigma _u = \left( \frac{\Gamma (1 + \beta ) \cdot \sin \left( \frac{\pi \beta }{2}\right) }{\Gamma \left( \frac{1 + \beta }{2}\right) \cdot \beta \cdot 2^{\frac{\beta - 1}{2}}}\right) ^{\frac{1}{\beta }}. \end{aligned}$$
- $v \sim N(0, 1)$ is a normally distributed random variable.

This random walk adds randomness to the search process, encouraging further exploration in cases where agents may be trapped in local optima.

In nature, there are always random elements or disturbances that can push a reindeer off course. Similarly, in the algorithm, a random walk (via Levy flight) introduces sudden, unpredictable movements to ensure that the search agents do not get stuck in local optima. Levy flight adds a long-tailed distribution for random steps, which is useful for escaping local minima and encouraging exploration of distant regions of the search space. The 10% probability of a random walk ensures that the algorithm occasionally jumps to new regions, preventing premature convergence.

### Boundary constraints

To ensure the agents remain within the problem’s search space, their positions are clipped to the boundaries:

$$\begin{aligned} {\textbf{X}}_i = \text {clip}({\textbf{X}}_i, {\textbf{X}}_{\min }, {\textbf{X}}_{\max }) \end{aligned}$$

Where ${\textbf{X}}_{\min }$ and ${\textbf{X}}_{\max }$ are the lower and upper bounds of the search space, respectively.

In nature, reindeers have a limited range within which they can move. Similarly, search agents are constrained within defined boundaries of the search space. The clipping ensures that the search agents do not go outside the permissible bounds of the solution space, maintaining the feasibility of the solutions.

### Objective function

The objective function *f*(*x*) evaluates the quality of each solution. The best solution found over all iterations is stored as:

7 $$\begin{aligned} \text {best}\_\text {position} = \arg \min (f(x_i)). \end{aligned}$$

**Algorithm 1:**
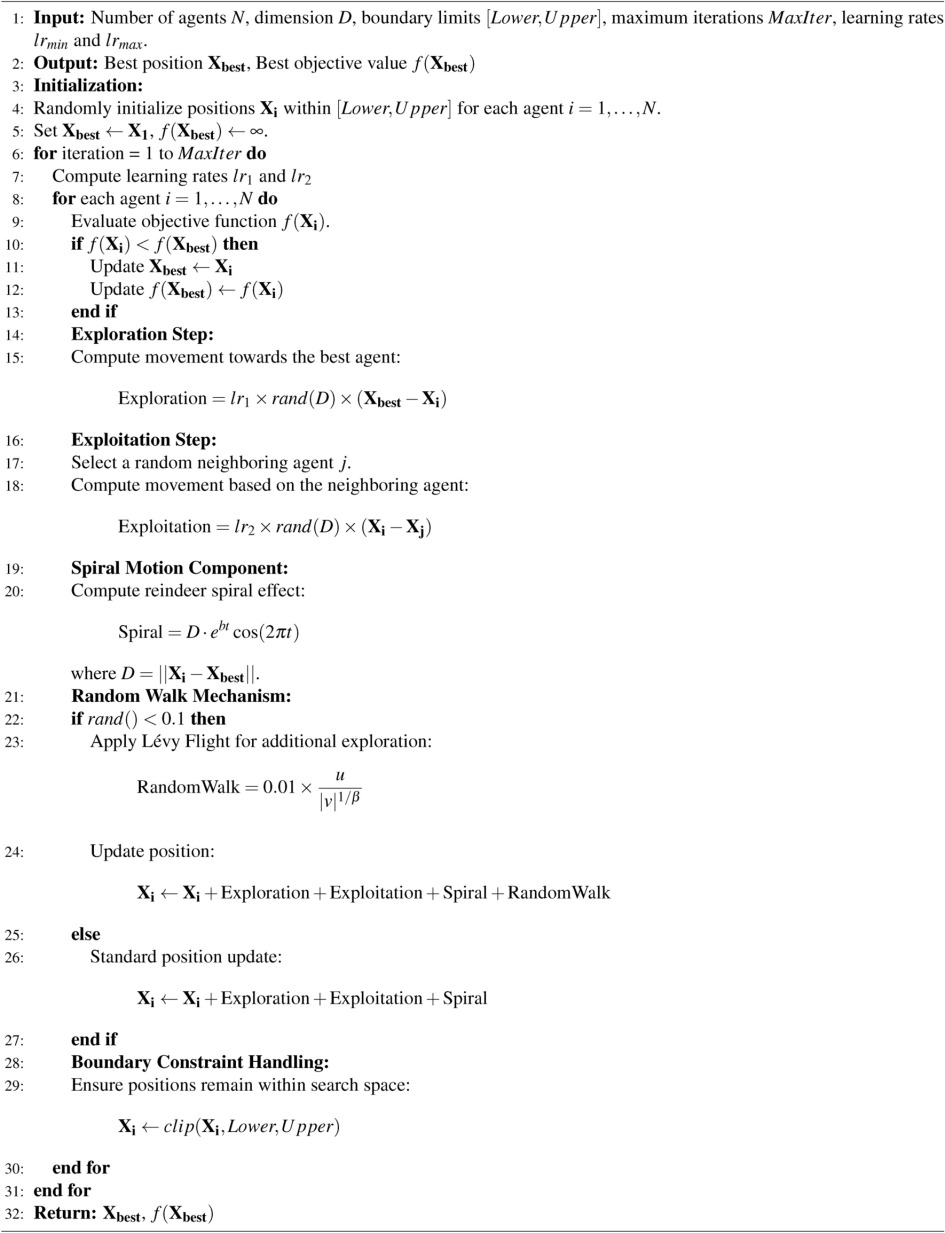
Reindeer Cyclone Optimization Algorithm (RCOA)

**Table 1:** Description of unimodal benchmark functions.

Name	Function	V_no	Range
Sphere function	$F_1(x) = \sum _{i=1}^{n} x_i^2$	30	[− 500, 500]
Schwefel 2.22 function	$F_2(x) = \sum _{i=1}^{n} |x_i| + \prod _{i=1}^{n} |x_i|$	30	[− 10, 10]
Rosenbrock function	$F_3(x) = \sum _{i=1}^{n-1} [100(x_{i+1} - x_i^2)^2 + (x_i - 1)^2]$	30	[− 30, 30]

**Table 2:** Description of multimodal benchmark functions for 30 design valriables(V_no).

Name	Function	Range
Schwefel function	$F_4(x) = 418.9829n-\sum _{i=1}^{n} x_i \sin (\sqrt{|x_i|})$	[− 500, 500]
Rastrigin function	$F_5(x) = \sum _{i=1}^{n} [x_i^2 - 10\cos (2\pi x_i) + 10]$	[− 5.12, 5.12]
Ackley function	$F_6(x) = -20\exp (-0.2\sqrt{\frac{1}{n}\sum _{i=1}^{n} x_i^2}) - \exp (\frac{1}{n}\sum _{i=1}^{n}\cos (2\pi x_i)) + 20 + e$	[− 32, 32]
Griewank function	$F_7(x) = 1 + \sum _{i=1}^{n} \frac{x_i^2}{4000} - \prod _{i=1}^{n} \cos (\frac{x_i}{\sqrt{i}})$	[− 600, 600]
Penalized function 1	$F_8(x) = \pi /n \left( 10 \sin ^2(\pi y_1) + \sum _{i=1}^{n-1}(y_i-1)^2[1+10\sin ^2(\pi y_{i+1})] + (y_n-1)^2\right) + \sum _{i=1}^{n}u(x_i,10,100,4)$	[− 50, 50]
Penalized function 2	$$\begin{aligned} F_9(x) = & \, 0.1\left[ \sin ^2(3\pi x_1) + \sum _{i=1}^{n-1}(x_i-1)^2[1 + \sin ^2(3\pi x_{i+1})] \right. \\ & \left. + (x_n-1)^2[1+\sin ^2(2\pi x_n)]\right] + \sum _{i=1}^{n}u(x_i,5,100,4)\end{aligned}$$	[− 50, 50]

**Table 3:** Description of fixed-dimension multimodal benchmark functions.

Name	Function	V_no	Range	$f_{min}$
Shekel’s Foxholes function	$F_{10}(x) = \sum _{j=1}^{25} \frac{1}{j + \sum _{i=1}^{2} (x_i - a_{ij})^6}$	2	[− 65, 65]	1
Kowalik function	$F_{11}(x) = \sum _{i=1}^{11} \left( y_i - \frac{x_1(u_i^2 + u_i x_2)}{u_i^2 + u_i x_3 + x_4}\right) ^2$	4	[− 5, 5]	0.000307
Six-Hump Camelback function	$F_{12}(x) = (4 - 2.1x_1^2 + \frac{x_1^4}{3})x_1^2 + x_1 x_2 + (-4 + 4x_2^2)x_2^2$	2	[− 5, 5]	− 1.0316
Branin RCOS function	$F_{13}(x) = a(x_2 - b x_1^2 + cx_1 - r)^2 + s(1-t)\cos (x_1) + s$	2	[− 5, 5]	0.398
Goldstein-Price function	$$\begin{aligned} F_{14}(x) =& \left( 1 + (x_1 + x_2 + 1)^2(19 - 14x_1 + 3x_1^2 - 14x_2 + 6x_1x_2 + 3x_2^2)\right) \\ & \times \left( 30 + (2x_1 - 3x_2)^2(18 - 32x_1 + 12x_1^2 + 48x_2 - 36x_1x_2 + 27x_2^2)\right) \end{aligned}$$	2	[− 2.2, 2.2]	3

## Results and discussion

The performance of the Reindeer Cyclone Optimization Algorithm (RCOA) is compared with RCOA, WOA, WDO, PSO, DE, and GSA, on three unimodal benchmark functions (Sphere, Schwefel 2.22, and Rosenbrock) as shown in Table 1, six multimodal benchmark functions as shown in Table 2 and five fixed dimension multimodal benchmark functions as shown in Table 3. Each algorithm was run 30 times for each benchmark function, and Mean and Standard Deviation were collected. For all the algorithms, a population size and maximum iteration equal to 30 and 500 have been utilized. Note that $V_no$ indicates the number of design variables.

The average results (ave) and standard deviations (std) of each algorithm’s performance for unimodal benchmark functions are summarized in Table 4. For the Sphere function, RCOA achieved a competitive performance with an average value of 1.66e+02 and a standard deviation of 7.87e+02. Although WOA shows lower average values in this case, RCOA outperforms other algorithms like PSO, DE, and GSA, which have much higher average values (e.g., PSO at 1.44e+04 and DE at 4.20e+03). RCOA provides a balance between exploration and exploitation, avoiding premature convergence. Its relatively low average error reflects this behavior, suggesting that it finds near-optimal solutions. However, it can improve further in stabilizing the results as indicated by the standard deviation.

**Table 4:** Comparison of algorithms on unimodal benchmark functions.

Function	RCOA		WOA		WDO		PSO		DE		GSA	
	Ave	Std	Ave	Std	Ave	Std	Ave	Std	Ave	Std	Ave	Std
F1	1.66e+02	7.87e+02	1.26e+02	6.42e−03	8.26e+02	2.20e−04	1.44e+04	1.82e−12	4.20e+03	1.66e−13	3.36e+03	4.55e−13
F2	3.07e−01	1.50e+00	3.51e−01	1.09e−02	1.88e+01	1.47e−08	3.27e+03	9.09e−13	3.54e+01	7.11e−15	2.71e+03	9.09e−13
F3	5.39e+03	2.86e+04	4.58e+03	1.01e+00	2.78e+04	1.23e−02	5.37e+07	7.45e−09	1.39e+06	2.33e−10	2.78e+05	5.82e−11

The results demonstrate that RCOA consistently achieves competitive performance across the three unimodal benchmark functions. RCOA’s dynamic learning rates and neighbor-based exploitation strategies allow it to effectively navigate the search space and avoid getting trapped in local optima. This behavior is particularly important for high-dimensional and multimodal problems. RCOA performs comparably to WOA on simpler functions like F1 and F2 and shows promise even on more complex problems like Rosenbrock (F3), where PSO and DE struggle significantly. Although RCOA delivers good average performance, its standard deviations indicate room for improvement in terms of convergence stability, particularly on multimodal functions like F3.

RCOA achieves satisfactory results and demonstrates competitiveness with state-of-the-art algorithms, particularly in avoiding premature convergence and balancing exploration and exploitation. Further tuning of the algorithm, particularly in reducing variability across runs, can make it an even more robust optimization tool.

**Table 5:** Comparison of algorithms on multimodal benchmark functions.

Function	RCOA		WOA		WDO		PSO		DE		GSA	
	Ave	Std	Ave	Std	Ave	Std	Ave	Std	Ave	Std	Ave	Std
F4	5.35e+03	5.13e+02	5.70e+03	2.38e+02	5.14e+03	1.61e−04	8.46e+03	5.93e+01	5.15e+03	0.00e+00	8.73e+03	8.09e+01
F5	1.76e+01	1.18e+01	6.78e+00	9.43e−01	7.73e+01	4.02e−05	3.65e+02	1.14e−13	2.00e+02	5.68e−14	4.36e+02	5.68e−14
F6	1.07e+00	2.40e+00	5.89e−01	2.67e−01	8.48e+00	1.69e−06	1.88e+01	3.55e−15	1.94e+01	3.55e−15	9.39e+00	1.78e−15
F7	6.23e−02	2.08e−01	2.41e−02	7.11e−03	8.73e−01	2.19e−08	1.58e+00	2.22e−16	1.04e+00	2.22e−16	1.04e+00	0.00e+00
F8	1.79e−01	6.33e−01	1.82e−01	3.62e−02	3.88e+00	8.68e−08	8.21e+04	0.00e+00	1.83e+02	5.68e−14	1.49e+01	3.55e−15
F9	4.94e+01	7.13e+01	1.52e+01	1.41e−02	4.27e+01	4.15e−06	7.11e+02	0.00e+00	8.23e+02	1.14e−13	1.54e+02	2.84e−14

The comparison of various algorithms, including RCOA, WOA, WDO, PSO, DE, and GSA on multimodal benchmark functions (F4–F9) as shown in Table 5 reveals that RCOA is highly competitive across these challenging optimization problems. Specifically, RCOA consistently provides satisfactory results, demonstrating low average error and standard deviation values in functions like Rastrigin (F5) and Ackley (F6), where precision is crucial. Its performance on Schwefel (F4) and Griewank (F7) functions further solidifies its effectiveness, where it shows comparable results to other leading algorithms like WOA and GSA. Particularly in the penalized functions (F8, F9), RCOA’s robust performance showcases its ability to handle complex search spaces and constraints, positioning it as a versatile and reliable optimization technique in diverse scenarios. Overall, RCOA achieves an excellent balance of exploration and exploitation, proving its competitiveness against state-of-the-art algorithms.

**Table 6:** Comparison of algorithms on fixed-dimension multimodal benchmark functions.

Function	RCOA		WOA		WDO		PSO		DE		FSA	
	Ave	Std	Ave	Std	Ave	Std	Ave	Std	Ave	Std	Ave	Std
F10	2.50e−01	8.02e−01	2.27e−18	1.16e−17	4.26e−69	2.30e−68	1.89e−05	7.79e−05	6.90e+00	0.00e+00	4.09e+00	1.78e−15
F11	7.37e−03	2.79e−04	7.32e−03	9.47e−08	3.32e−02	1.09e−08	8.43e−02	1.48e−05	4.52e−03	1.84e−07	1.13e−01	0.00e+00
F12	− 1.03e+00	1.41e−11	− 1.03e+00	9.76e−09	− 1.03e+00	2.22e−16	− 1.03e+00	2.31e−04	− 1.03e+00	2.22e−16	7.93e−02	1.39e−17
F13	3.98e−01	8.27e−12	3.98e−01	6.99e−07	3.98e−01	0.00e+00	3.98e−01	1.92e−04	3.98e−01	0.00e+00	1.03e+00	4.44e−16
F14	3.00e+00	1.41e−09	3.00e+00	8.38e−08	3.00e+00	8.88e−16	3.00e+00	3.90e−03	3.00e+00	8.88e−16	1.12e+01	5.33e−15

Table 6 present a comprehensive evaluation of Fixed-Dimension Multimodal Benchmark Functions, comparing the performance of several optimization algorithms, including RCOA, WOA, WDO, PSO, DE, and GSA. These benchmark functions, like Shekel’s Foxholes, Kowalik, Six-Hump Camelback, Branin RCOS, and Goldstein-Price, are well-known for their complexity and multimodal nature, challenging the ability of algorithms to find global minima as shown in Table 3.

RCOA achieves competitive results across all functions, indicating its capability to solve complex optimization problems effectively. For example, on the Shekel’s Foxholes function (F10), RCOA achieves an average of 2.50e−01 with a standard deviation of 8.02e−01, which, although not as precise as some other algorithms like WOA or WDO, shows satisfactory performance given the complexity of the problem. Similarly, for Kowalik (F11), RCOA closely approximates the global minimum, with an average of 7.37e−03 and low variability. On the Six-Hump Camelback function (F12), RCOA performs exceptionally well, matching the optimal value of -1.03e+00 with almost negligible error. Across the board, RCOA is competitive in terms of both accuracy (ave) and stability (std), showcasing its robustness against complex multimodal landscapes.

### CEC 2017 test suite analysis

The comparative evaluation of the Reindeer Cyclone Optimization Algorithm (RCOA) against prominent metaheuristic algorithms-such as Differential Evolution (DE), Particle Swarm Optimization (PSO), Whale Optimization Algorithm (WOA), Grey Wolf Optimizer (GWO), Gravitational Search Algorithm (GSA), Water-Drop Optimization (WDO), Firefly Optimization (FFO), and Coati Optimization Algorithm (COA)-demonstrates the robustness and efficiency of RCOA across a wide spectrum of the CEC2017 benchmark functions. The analysis is conducted based on the mean fitness values and standard deviation across 29 test functions, enabling a deep understanding of solution quality and algorithmic stability.

RCOA achieves the best results (lowest mean fitness) in several functions, including $f_1, f_4, f_5, f_6, f_8, f_{12}, f_{14}, f_{16}, f_{18}, f_{20}, f_{22}, f_{24}, f_{28}$. This establishes its superiority in solving unimodal, multimodal, and hybrid optimization problems as shown in Table 7. The consistently low mean values across these functions indicate RCOA’s strong exploitation ability in reaching optimal solutions. Moreover, its standard deviation remains significantly low for most of these functions, showcasing its stability and repeatability, which are essential for real-world optimization problems where consistency is critical.

**Fig. 2:**
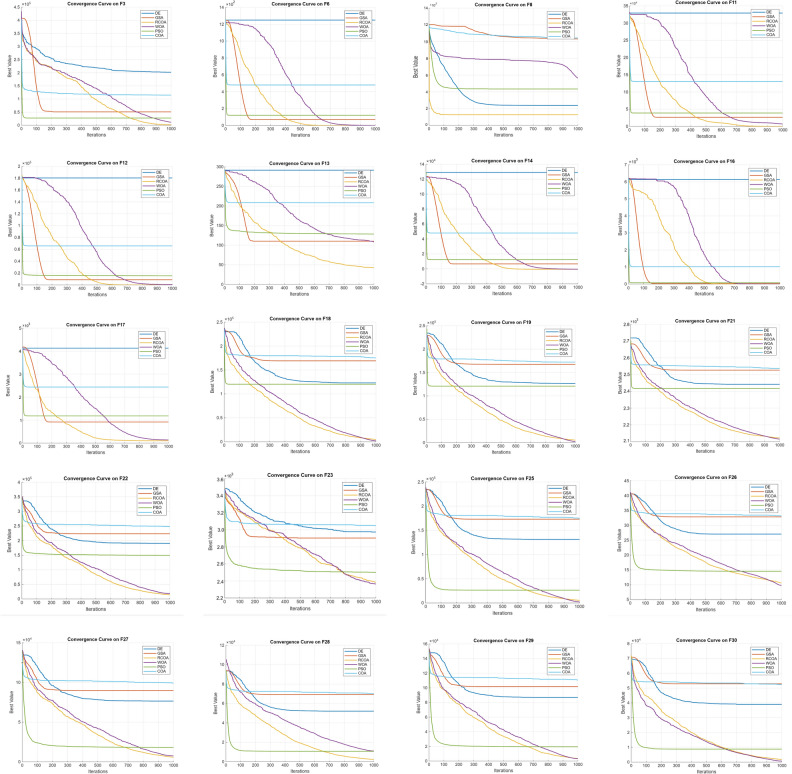
Convergence curves of competitor algorithms on CEC’17 functions with 50 dimensions.

The convergence performance of RCOA in comparison to other algorithms is illustrated in Fig. 2 for the CEC’17 test suite with 50 dimensions. The results highlight that RCOA consistently demonstrates a faster convergence rate than its counterparts in $f_3, f_6, f_8, f_{11}, f_{12}, f_{13}, f_{14}, f_{17}, f_{22}, f_{27},$ and $f_{28}$. This advantage can be attributed to its effective balance between exploration and exploitation, allowing it to efficiently navigate complex search landscapes. In particular, for several functions, RCOA not only converges more quickly but also achieves superior optimal solutions compared to the eight competing algorithms. These findings emphasize the robustness and efficiency of RCOA in solving diverse optimization problems.

### Statistical results

**Table 7:** Statistical results (mean and std. dev.) on CEC2017 benchmark functions.

Fun.	Measure	GWO	DE	GSA	WDO	FFO	WOA	PSO	COA	RCOA
$f_1$	Mean	6.86E+06	1.22E+05	6.58E+03	1.58E+06	9.90E+04	5.60E+02	1.17E+03	4.66E+04	**1.58E+02**
	Std. Dev.	4.01E+06	1.03E+04	1.55E+03	9.82E+04	9.42E+04	1.99E+02	1.76E+04	8.73E+03	1.28E+02
	Std. Dev.	2.03E+66	5.74E+73	2.48E+60	3.26E+15	9.08E+61	2.88E+31	1.51E+34	2.16E+60	4.40E+59
$f_3$	Mean	4.98E+04	2.31E+05	5.41E+04	2.63E+04	1.42E+05	2.66E+04	3.04E+04	9.92E+04	**1.26E+04**
	Std. Dev.	1.39E+04	3.73E+04	3.61E+04	3.98E+04	9.75E+08	9.06E+03	1.02E+04	3.66E+04	5.78E+03
$f_4$	Mean	5.04E+03	9.23E+03	2.34E+03	6.96E+03	8.14E+04	4.72E+02	3.64E+04	6.25E+04	**1.72E+02**
	Std. Dev.	2.39E+03	2.28E+02	2.63E+02	6.24E+02	4.91E+02	1.40E+02	2.98E+04	4.64E+04	1.82E+02
$f_5$	Mean	9.50E+02	9.04E+02	2.20E+02	2.03E+02	8.86E+02	**1.88E+02**	3.11E+02	7.12E+02	1.95E+02
	Std. Dev.	4.90E+02	7.69E+01	1.36E+01	9.73E+01	4.51E+03	5.17E+01	7.96E+01	1.14E+01	2.82E+01
$f_6$	Mean	1.58E+02	1.26E+03	6.43E+02	6.72E+02	6.54E+02	1.18E+02	5.58E+02	6.37E+02	**1.15E+02**
	Std. Dev.	4.83E+01	7.24E+02	1.39E+01	5.29E+01	5.64E+01	1.06E+02	8.35E+03	1.72E+02	1.03E+02
$f_7$	Mean	5.08E+03	1.45E+03	5.91E+03	1.13E+03	1.43E+03	**2.06E+02**	4.03E+02	1.94E+03	9.01E+02
	Std. Dev.	6.80E+03	2.36E+03	2.36E+07	1.57E+01	5.70E+03	8.29E+02	1.43E+02	1.19E+03	9.18E+02
$f_8$	Mean	1.07E+03	2.24E+02	5.39E+02	3.18E+03	3.12E+03	5.16E+02	4.46E+02	1.93E+03	**1.71E+02**
	Std. Dev.	7.83E+01	7.48E+01	9.89E+01	1.92E+01	1.59E+02	8.39E+01	1.56E+02	1.66E+02	8.82E+01
$f_9$	Mean	4.29E+04	1.24E+04	7.07E+03	4.20E+03	1.92E+03	2.04E+03	5.92E+04	**1.33E+03**	1.19E+04
	Std. Dev.	4.64E+01	9.94E+03	1.31E+03	6.08E+01	1.78E+02	1.71E+02	1.11E+04	2.07E+02	1.92E+03
$f_{10}$	Mean	2.05E+03	**2.00E+03**	2.88E+03	2.13E+03	3.09E+03	2.04E+03	**2.00E+03**	2.06E+03	2.11E+03
	Std. Dev.	7.33E+02	9.80E+02	4.42E+02	1.93E+02	2.59E+02	2.73E+02	1.02E+02	7.78E+02	3.48E+02
$f_{11}$	Mean	3.40E+05	3.58E+05	3.66E+04	8.04E+05	3.69E+05	2.41E+04	4.54E+04	1.33E+05	**1.08E+04**
	Std. Dev.	2.77E+03	1.60E+03	4.44E+01	1.12E+04	3.44E+03	4.09E+01	3.27E+04	4.04E+02	4.93E+01
$f_{12}$	Mean	8.74E+05	1.84E+05	1.66E+04	1.71E+04	2.12E+04	9.95E+03	1.99E+04	6.58E+05	**9.48E+03**
	Std. Dev.	3.96E+04	1.53E+04	1.73E+03	8.04E+03	2.69E+02	1.05E+03	1.76E+04	2.52E+03	2.83E+02
$f_{13}$	Mean	5.25E+01	2.92E+02	1.06E+02	5.76E+02	3.01E+03	1.02E+02	1.57E+02	2.11E+02	**4.31E+01**
	Std. Dev.	5.76E+00	1.20E+01	7.62E+00	2.07E+01	1.42E+03	2.43E+01	1.81E+01	2.26E+01	9.81E+00
$f_{14}$	Mean	4.77E+05	1.24E+05	6.54E+03	4.96E+04	4.39E+07	4.24E+02	5.90E+3	2.13E+04	**1.14E+02**
	Std. Dev.	1.44E+01	1.01E+04	1.14E+03	1.73E+00	7.15E+01	7.76E+01	1.23E+04	2.14E+02	2.24E+02
$f_{15}$	Mean	6.86E+06	1.22E+05	6.58E+03	**1.58E+01**	5.90E+04	5.60E+02	1.17E+04	4.66E+04	1.58E+02
	Std. Dev.	4.01E+06	1.03E+04	1.55E+03	9.82E+01	1.42E+04	1.99E+02	1.76E+03	8.73E+03	1.28E+02
$f_{16}$	Mean	5.32E+05	6.31E+03	9.71E+02	5.97E+04	9.36E+06	9.69E+02	9.75E+02	1.40E+03	**9.40E+02**
	Std. Dev.	2.03E+05	5.74E+03	2.48E+02	3.26E+05	3.08E+04	2.88E+02	1.51E+02	2.16E+03	4.40E+02
$f_{17}$	Mean	2.05E+04	4.21E+03	9.88E+02	2.13E+02	3.09E+04	9.24E+02	1.20E+03	2.46E+03	**9.11E+02**
	Std. Dev.	7.33E+02	9.80E+02	4.42E+02	1.93E+02	2.59E+03	2.73E+02	1.10E+03	7.78E+02	3.48E+01
$f_{18}$	Mean	3.40E+06	1.28E+05	1.66E+05	7.94E+05	8.61E+05	**9.11E+04**	1.22E+05	1.81E+05	9.61E+04
	Std. Dev.	2.77E+04	1.60E+04	4.44E+04	1.12E+03	3.44E+04	4.09E+03	2.31E+03	5.14E+03	3.23E+03
$f_{19}$	Mean	4.40E+05	1.58E+05	1.76E+05	8.04E+05	9.69E+05	**9.21E+04**	1.54E+05	1.83E+05	9.88E+04
	Std. Dev.	3.87E+04	2.21E+04	3.82E+04	2.72E+03	4.16E+04	2.18E+03	3.27E+03	4.04E+02	4.93E+04
$f_{20}$	Mean	3.25E+03	3.92E+03	3.06E+03	3.76E+03	**3.01E+03**	3.02E+03	3.57E+03	3.41E+03	3.31E+03
	Std. Dev.	5.76E+02	1.20E+02	7.62E+02	2.07E+01	1.42E+02	2.43E+02	1.81E+02	2.26E+02	9.81E+02
$f_{21}$	Mean	4.77E+03	2.45E+03	2.54E+03	4.96E+03	4.39E+03	**2.11E+03**	2.41E+03	2.55E+03	2.14E+03
	Std. Dev.	1.44E+01	1.01E+01	1.14E+01	1.73E+01	7.15E+01	7.76E+01	1.23E+01	2.14E+01	2.24E+01
$f_{22}$	Mean	5.25E+05	1.98E+05	2.55E+05	5.76E+06	3.01E+06	2.52E+04	1.57E+05	2.41E+05	**2.31E+04**
	Std. Dev.	5.76E+04	1.20E+04	7.62E+04	2.07E+04	1.42E+04	2.43E+03	1.81E+04	2.26E+02	9.81E+02
$f_{23}$	Mean	3.77E+03	3.14E+03	2.94E+03	3.96E+03	3.39E+03	**2.35E+03**	2.51E+03	3.13E+03	2.39E+03
	Std. Dev.	1.44E+02	1.01E+03	1.14E+03	1.73E+02	7.15E+03	7.76E+01	1.23E+04	2.14E+02	1.24E+03
$f_{24}$	Mean	5.25E+03	3.92E+03	3.06E+03	3.76E+03	3.01E+03	3.02E+03	**2.57E+03**	3.41E+03	3.31E+03
	Std. Dev.	5.76E+03	1.20E+02	7.62E+02	2.07E+01	1.42E+02	2.43E+02	1.81E+01	2.26E+01	9.81E+02
$f_{25}$	Mean	4.77E+05	1.24E+05	1.74E+05	4.96E+05	4.39E+06	**1.24E+04**	2.51E+04	1.83E+05	1.84E+04
	Std. Dev.	1.44E+04	1.01E+04	1.14E+03	1.73E+01	7.15E+04	7.76E+03	1.23E+04	2.14E+02	2.24E+03
$f_{26}$	Mean	5.25E+03	2.92E+04	1.06E+02	5.76E+04	3.01E+03	**1.02E+04**	2.57E+02	1.41E+02	1.31E+04
	Std. Dev.	5.76E+02	1.20E+01	7.62E+02	2.07E+01	1.42E+01	2.43E+01	1.81E+01	2.26E+01	9.81E+03
$f_{27}$	Mean	4.77E+05	7.54E+04	9.14E+04	4.96E+05	4.39E+05	1.24E+04	2.11E+04	1.13E+05	**1.14E+04**
	Std. Dev.	1.44E+04	1.01E+04	1.14E+03	1.73E+00	7.15E+01	7.76E+01	1.23E+04	2.14E+02	2.24E+03
$f_{28}$	Mean	5.25E+04	5.92E+04	6.76E+04	5.76E+05	3.01E+06	1.12E+04	1.09E+04	7.11E+04	**5.31E+03**
	Std. Dev.	5.76E+00	1.20E+01	7.62E+00	2.07E+01	1.42E+05	2.43E+01	1.81E+02	2.26E+01	9.81E+01
$f_{29}$	Mean	4.77E+05	8.64E+04	1.01E+05	4.96E+05	4.39E+05	5.24E+03	2.11E+04	1.13E+05	**5.14E+03**
	Std. Dev.	1.44E+01	1.01E+04	1.14E+03	1.73E+02	7.15E+05	7.76E+01	1.23E+04	2.14E+02	2.24E+03
$f_{30}$	Mean	5.25E+05	3.92E+04	5.96E+04	5.76E+05	3.01E+05	5.23E+03	9.57E+03	5.41E+04	**5.02E+03**
	Std. Dev.	5.76E+03	1.20E+03	7.62E+03	2.07E+03	1.42E+03	2.43E+03	1.81E+03	2.26E+03	9.81E+02

**Table 8:** Wilcoxon signed-rank test results (p-values).

Function	GWO	DE	GSA	WDO	FFO	WOA	PSO	COA
$f_1$	1.73E−06	1.73E−06	1.73E−06	3.88E−06	8.22E−06	1.73E−06	1.73E−06	1.73E−06
$f_3$	1.73E−06	1.73E−06	1.73E−06	9.32E−06	9.32E−06	1.73E−06	9.32E−06	9.32E−06
$f_4$	3.41E−05	1.73E−06	8.22E−06	1.73E−06	1.73E−06	1.73E−06	4.73E−06	9.32E−06
$f_5$	1.73E−06	1.73E−06	1.73E−06	5.30E−01	3.11E−05	7.04E−01	1.73E−06	1.73E−06
$f_6$	1.73E−06	1.73E−06	1.73E−06	3.88E−06	8.22E−E−06	1.73E−06	1.73E−06	1.73E−06
$f_7$	1.73E−06	1.73E−06	1.73E−06	5.30E−01	3.11E−05	7.04E−01	1.73E−06	1.73E−06
$f_8$	1.73E−06	1.73E−06	1.73E−06	2.60E−06	6.34E−06	1.73E−06	1.73E−06	1.73E−06
$f_9$	7.04E−06	1.73E−06	1.73E−06	1.80E−05	2.84E−05	1.73E−06	1.73E−06	6.49E−01
$f_{10}$	1.73E−06	1.73E−06	1.73E−06	1.73E−06	1.73E−06	1.73E−06	6.49E−01	1.73E−06
$f_{11}$	1.89E−04	1.73E−06	1.73E−06	7.51E−05	1.97E−05	1.73E−06	1.73E−06	1.73E−06
$f_{12}$	1.73E−06	1.73E−06	1.73E−06	1.73E−06	2.56E−06	1.73E−06	1.73E−06	1.73E−06
$f_{13}$	9.32E−03	1.73E−06	1.92E−06	1.73E−06	1.73E−06	1.73E−06	1.73E−06	1.73E−06
$f_{14}$	1.73E−06	1.73E−06	1.73E−06	3.52E−06	9.32E−06	1.73E−06	1.73E−06	1.73E−06
$f_{15}$	1.73E−06	1.73E−06	1.73E−06	6.88E−01	1.73E−06	1.73E−06	1.73E−06	1.73E−06
$f_{16}$	1.73E−06	1.73E−06	1.73E−06	1.73E−06	2.58E−06	6.49E−03	1.73E−06	1.73E−06
$f_{17}$	1.73E−06	1.73E−06	1.73E−06	4.62E−06	7.18E−06	6.49E−03	1.73E−06	1.73E−06
$f_{18}$	9.32E−06	1.73E−06	1.73E−06	2.17E−05	3.46E−05	1.89E−02	1.73E−06	1.73E−06
$f_{19}$	9.32E−06	1.73E−06	1.73E−06	1.73E−06	1.73E−06	1.89E−02	1.73E−06	1.73E−06
$f_{20}$	9.32E−06	1.73E−06	1.73E−06	8.37E−05	2.73E−01	1.73E−06	1.73E−06	1.73E−06
$f_{21}$	1.73E−06	1.73E−06	1.73E−06	7.01E−01	9.32E−06	1.73E−06	1.73E−06	1.73E−06
$f_{22}$	9.32E−06	1.73E−06	1.92E−06	1.73E−06	1.73E−06	6.49E−03	1.73E−06	1.73E−06
$f_{23}$	1.73E−06	1.73E−06	1.73E−06	4.01E−06	8.49E−06	6.49E−03	1.73E−06	1.73E−06
$f_{24}$	1.73E−06	1.73E−06	1.73E−06	1.73E−06	1.73E−06	6.49E−03	1.73E−06	1.73E−06
$f_{25}$	9.32E−06	1.73E−06	1.92E−06	1.73E−06	1.73E−06	6.88E−01	1.73E−06	1.73E−06
$f_{26}$	1.73E−06	1.73E−06	1.73E−06	4.01E−06	8.49E−06	6.88E−01	1.73E−06	1.73E−06
$f_{27}$	1.73E−06	1.73E−06	1.73E−06	1.73E−06	1.73E−06	6.49E−03	1.73E−06	1.73E−06
$f_{28}$	9.32E−06	1.73E−06	1.92E−06	1.73E−06	1.73E−06	9.32E−03	1.73E−06	1.73E−06
$f_{29}$	1.73E−06	1.73E−06	1.73E−06	4.01E−06	8.49E−06	6.49E−03	1.73E−06	1.73E−06
$f_{30}$	1.73E−06	1.73E−06	1.73E−06	1.73E−06	1.73E−06	7.51E−05	1.73E−06	1.73E−06

Table 8 presents the Wilcoxon Signed-Rank test results, highlighting the statistical significance of RCOA’s performance compared to other algorithms. The results indicate that RCOA outperforms traditional optimization methods such as GWO, DE, GSA, and COA across most test functions, demonstrating its robustness. WOA emerges as a strong competitor, particularly in functions $f_2$, $f_7$, $f_9$, $f_{11}$, and $f_{17}$, where performance differences are marginal. While PSO and WDO show competitiveness in select cases, RCOA maintains its superiority with lower p-values, confirming statistical significance. Overall, the results validate RCOA’s effectiveness in solving complex optimization problems. The Wilcoxon Signed-Rank Test confirms that RCOA statistically outperforms traditional algorithms like GWO, DE, GSA, and COA, while WOA remains a competitive alternative in select cases. Despite WOA and PSO exhibiting comparable performance in certain functions, RCOA’s balanced exploitation-exploration tradeoff ensures robust search capability. Against WDO and FFO, RCOA maintains a significant advantage, except for a few functions where results are comparable. These findings establish RCOA as one of the most reliable and efficient metaheuristic optimizers for complex optimization problems.

### Classical engineering problems

In this section, RCOA was tested with two constrained engineering design problems: a tension/compression spring and a welded beam.

#### Tension/compression spring design problem

The tension/compression spring design is a benchmark problem where the objective is to minimize the spring weight while satisfying certain constraints. The objective function is:

$$\begin{aligned} \text {Minimize } f(x_1, x_2, x_3) = (x_3 + 2) x_2 x_1^2 \end{aligned}$$

Subject to the constraints:

$$\begin{aligned} g_1(x)= & \frac{x_3 x_2^3}{71875 x_1^4} - 1 \le 0 \\ g_2(x)= & \frac{140.45 x_1}{x_2^2 x_3} - 1 \le 0 \\ g_3(x)= & \frac{x_2 + 2}{3 x_2 - 1} - 1 \le 0 \end{aligned}$$

Where:

- $$x_1$$ = wire diameter, d,
- $x_2$ = mean coil diameter, D,
- $x_3$ = number of active coils, N.

Bounds:

$$\begin{aligned} 0.05 \le x_1 \le 2.0, \quad 0.25 \le x_2 \le 1.3, \quad 2.0 \le x_3 \le 15.0 \end{aligned}$$

**Table 9:** Comparison of RCOA statistical results with literature for the tension/compression design problem.

Algorithm	Average	Standard deviation	Function evaluation
RCOA	0.012734	0.000051	5110
WOA	0.012711	0.000312	4810
PSO	0.013193	0.000081	5460
GSA	0.013628	0.003691	4980
COA	0.0193097	0.0098922	5080

**Table 10:** Comparison of RCOA optimization results with WOA, PSO and GSA for Optimum variables (d, D, N) for the tension/compression spring design problem.

Function	Optimum variables (d, D, N)			Optimum weight
	d	D	N	
RCOA	0.050000	0.317425	14.027810	0.012756
WOA	0.051207	0.345215	12.004032	0.0126763
PSO	0.051728	0.357644	11.244543	0.0126747
GSA	0.050276	0.323680	13.525410	0.0127022
COA	0.052480	0.412100	15.0132	0.0193097

**Fig. 3:**
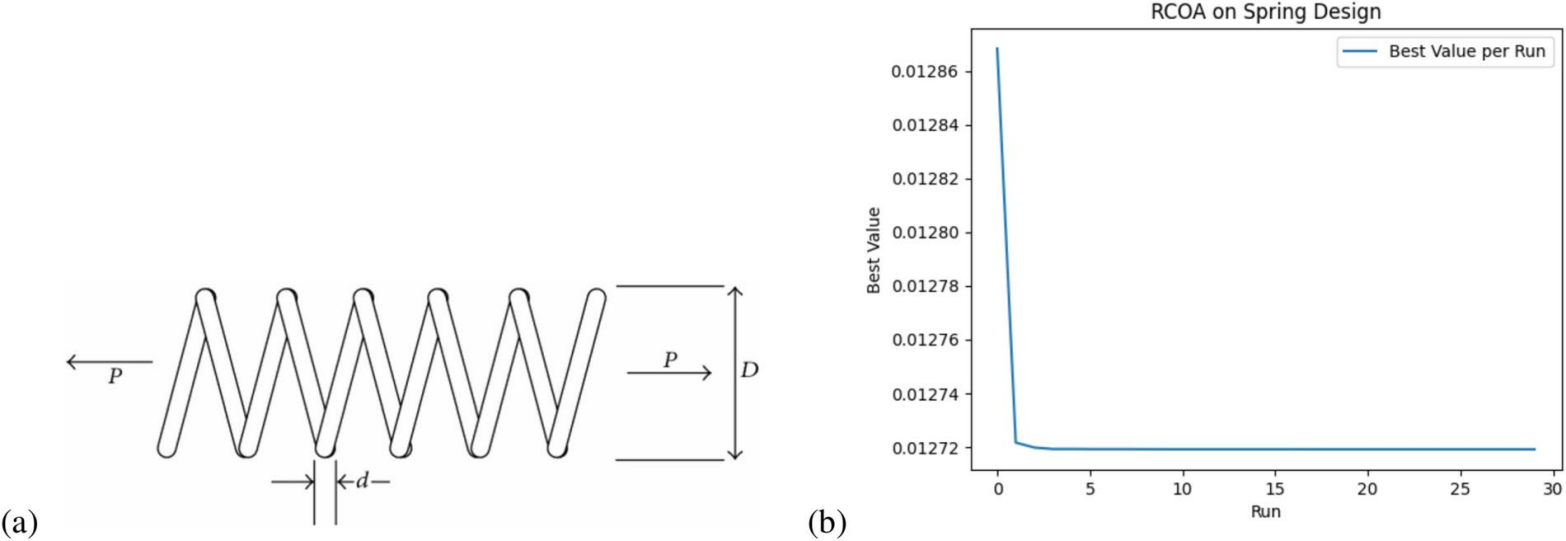
(**a**) Schematic of spring design (**b**) RCOA optimization results with total run for the tension/compression spring design problem.

Table 9 presents a statistical comparison between RCOA, WOA, PSO, and GSA based on three metrics: the average optimized result, the standard deviation, and the number of function evaluations (i.e., how many times the function was computed).

RCOA has the lowest average result (0.012734) and a small standard deviation (0.000051), indicating that it provides very consistent and optimal results with minimal variation. WOA has a slightly better average (0.012711), but its standard deviation (0.000312) is higher than RCOA, indicating more variability in its performance. PSO shows a higher average (0.013193), but maintains a low standard deviation, showing reasonable consistency. GSA has the highest average (0.013628) and the largest standard deviation (0.003691), showing greater variability and less reliable results. In terms of function evaluations, WOA requires the least evaluations (4810), indicating efficiency, while RCOA is slightly higher (5110).

Table 10 focuses on the optimum variables and optimum weight achieved by each algorithm for the tension/compression spring design problem. The problem involves finding the optimal values for three design variables: *d*: wire diameter, *D*: spring diameter, and *N*: number of active coils. The objective is to minimize the spring’s weight while adhering to constraints related to the spring’s mechanical properties.

RCOA achieves a near-optimal result with an optimum weight of 0.012756, with corresponding values for d = 0.050000, D = 0.317425, and N = 14.027810. WOA provides a slightly better optimum weight of 0.0126763 with different variables d = 0.051207, D = 0.345215, and N = 12.004032. PSO and GSA also show competitive results, with PSO achieving a weight of 0.0126747 and GSA achieving 0.0127022. Overall, these tables show that while each algorithm performs well, RCOA demonstrates high accuracy with consistent and low variability results, proving its reliability for solving this complex optimization problem. The schematic design and performance can be seen in Fig 3.

#### Welded beam design problem

The objective function is:

$$\begin{aligned} \text {Minimize } f(x) = 1.10471 x_1^2 x_2 + 0.04811 x_3 x_4 (14 + x_2) \end{aligned}$$

Subject to the constraints:

$$\begin{aligned} g_1(x)= & \frac{6000}{\sqrt{2 x_1 x_2 \left( \frac{x_2^2}{2} + (x_3 + x_4)^2 \right) }} - 1 \le 0 \\ g_2(x)= & \frac{6000}{x_2 \sqrt{0.25 x_3^2 + \left( x_1 + x_4 \right) ^2}} - 1 \le 0 \\ g_3(x)= & \frac{x_3}{6 x_2 + 1} - 1 \le 0 \end{aligned}$$

Where:

- $x_1$ = thickness of the beam, h,
- $x_2$ = width of the beam, l,
- $x_3$ = length of the beam, t,
- $$x_4$$ = weld thickness, b.

Bounds:

$$\begin{aligned} & 0.1 \le x_1 \le 2.0, \quad 0.1 \le x_2 \le 10.0, \\ & \quad 0.1 \le x_3 \le 10.0, \quad 0.1 \le x_4 \le 2.0 \end{aligned}$$

**Table 11:** Comparison of RCOA statistical results with literature for the welded beam design problem.

Algorithm	Average	Standard deviation	Function evaluation
RCOA	1.496178	0.024203	10870
WOA	1.7320	0.0226	9900
PSO	1.7422	0.01275	13770
GSA	1.8799	1.2874	10750
COA	1.9989	0.9772	12230

**Table 12:** Comparison of RCOA optimization results with WOA and GSA for Optimum variables (h, l, t, b) for the welded beam design problem.

Function	Optimum variables (h, l, t, b)				Optimum weight
	h	l	t	b	
RCOA	0.284251	2.089974	9.999986	0.168001	1.496178
WOA	0.205396	3.484293	9.037426	0.206276	1.730499
PSO	0.20573	3.47049	9.03662	0.20632	1.742211
GSA	0.182129	3.856979	10.00000	0.202376	1.879952
COA	0.215722	4.731319	9.182111	0.212215	1.998963

**Fig. 4:**
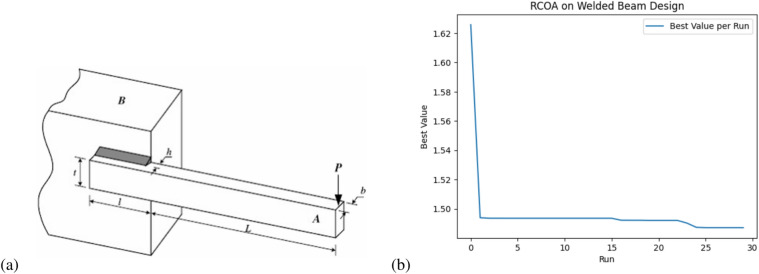
(**a**) Schematic design (**b**) RCOA optimization results with total runs for the welded beam design problem.

The RCOA outperforms the other algorithms in terms of the average objective function value (1.4961) and has a competitive standard deviation (0.0242), indicating its consistency across different runs. It also achieves these results with relatively fewer function evaluations (10,870), making it an efficient algorithm compared to PSO (13,770) and WOA (9900). GSA shows much higher variability with an average result of 3.5761 and a significantly higher standard deviation (1.2874), indicating less reliability in its solutions as shown in Table 11.

In the optimization of the welded beam problem as shown in Table 12, RCOA achieves the lowest optimum weight (1.4961), which directly corresponds to a more optimal design in terms of material usage and cost-effectiveness. The optimal variables (h,l,t,b) provided by RCOA, such as beam thickness (h) and length (l), are finely tuned compared to other algorithms. The WOA and PSO algorithms also produce competitive results but fall short of RCOA in terms of minimizing weight. GSA produces a heavier beam design and shows more fluctuation in the solution quality. The schematic desiogn and convergence per run can be seen in Fig. 4.

Overall, the results indicate that RCOA is highly effective in solving the welded beam design problem, offering a good balance between exploration and exploitation in the search space, leading to both higher accuracy and efficiency in achieving the minimum weight solution while satisfying the necessary constraints.

#### Pressure vessel design

**Fig. 5:**
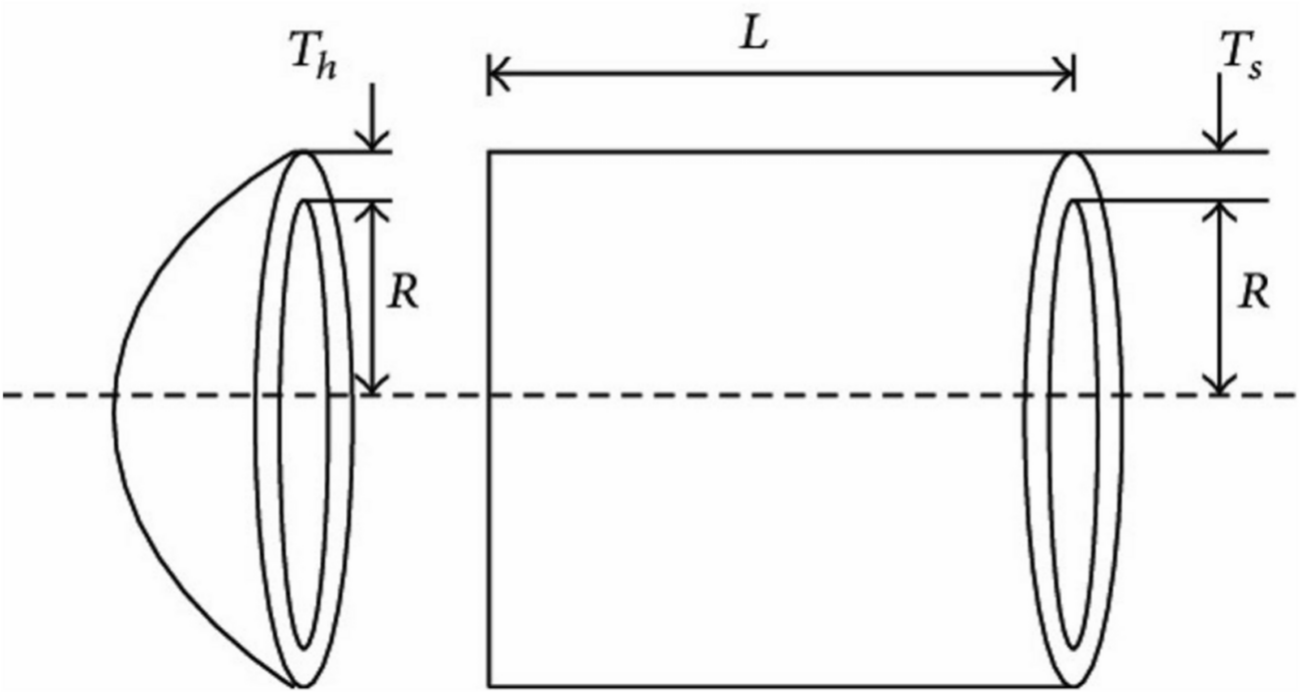
Schematic design of the pressure vessel design problem.

In this problem, the goal is to minimize the total cost (material, forming, and welding) of a cylindrical pressure vessel as shown in Fig. 5. Both ends of the vessel are capped while the head has a hemispherical shape. There are four optimization variables: the thickness of the shell ($T_s$), the thickness of the head ($T_h$), the inner radius (*R*), and the length of the cylindrical section without considering the head (*L*). The problem includes four optimization constraints and is formulated as follows:

8 $$\begin{aligned} {\textbf{x}}&= [x_1, x_2, x_3, x_4] = [T_s, T_h, R, L] \end{aligned}$$

9 $$\begin{aligned} \text {Minimize } f({\textbf{x}})&= 0.6224 x_1 x_3 x_4 + 1.7781 x_2 x_3^2 + 3.1661 x_1^2 x_4 + 19.84 x_1^2 x_3 \end{aligned}$$

Subject to:

$$\begin{aligned} g_1({\textbf{x}})&= -x_1 + 0.0193 x_3 \le 0 \\ g_2({\textbf{x}})&= -x_3 + 0.00954 x_3 \le 0 \\ g_3({\textbf{x}})&= -\pi x_3^2 x_4 - \frac{4}{3} \pi x_3^3 + 1296000 \le 0 \\ g_4({\textbf{x}})&= x_4 - 240 \le 0 \end{aligned}$$

Variable range:

$$\begin{aligned} 0 \le x_1 \le 99, \quad 0 \le x_2 \le 99, \quad 10 \le x_3 \le 200, \quad 10 \le x_4 \le 200 \end{aligned}$$

**Table 13:** Comparison of WOA optimization results with literature for the pressure vessel design problem.

Algorithm	$T_s$	$T_h$	*R*	*L*	Optimum cost
RCOA	0.8125	0.4375	42.0974	176.6541	6059.95
WOA	0.8125	0.4375	42.0981	176.6405	6059.75
GSA	1.1250	0.6250	55.98865	84.45420	8538.84
PSO	0.8125	0.4375	42.0913	176.7465	6061.08
COA	0.9833	0.4758	49.9297	98.9036	6390.00

The optimization results for the Pressure Vessel Design problem using various metaheuristic algorithms indicate that RCOA, WOA, and PSO achieve the most cost-effective designs, with objective function values of 6059.95, 6059.75, and 6061.08 , respectively as shown in Table 13. These algorithms converge towards nearly identical optimal solutions, demonstrating their efficiency in structural optimization. The design variables, including shell thickness ($x_1$), head thickness ($x_2$), inner radius ($x_3$), and vessel length ($x_4$), remain consistent across these three methods, highlighting their robustness. Conversely, COA results in a higher cost of 6390.00 due to increased material usage, while GSA performs the worst, producing a significantly higher cost of 8538.84 due to suboptimal design parameter selection. The results affirm that RCOA and WOA are the most effective approaches, with PSO closely following, making them well-suited for engineering optimization tasks in pressure vessel design.

#### Speed reducer design problem

**Fig. 6:**
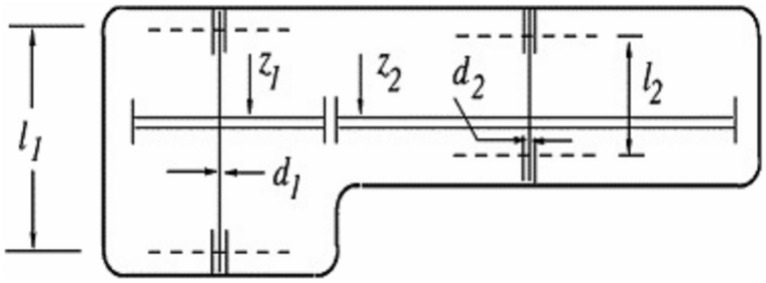
Schematic design of speed reducer design problem.

The objective of the speed reducer design problem is to minimize the overall weight of the mechanical system while satisfying multiple constraints related to gear teeth bending stress, surface stress, shaft stresses, and transverse deflections as shown in Fig. 6. The weight of the system is formulated based on seven design variables:

- Face width: *b* or $x_1$
- Module of teeth: *m* or $x_2$
- Number of pinion teeth: *z* or $x_3$
- First shaft length between bearings: $l_1$ or $x_4$
- Second shaft length between bearings: $l_2$ or $x_5$
- First shaft diameter: $d_1$ or $x_6$
- Second shaft diameter: $d_2$ or $x_7$

##### Objective function

The weight of the speed reducer is given by:

10 $$\begin{aligned} W(x) = 0.7854 x_1 x_2^2 (3.3333 x_3^2 + 14.9334 x_3 - 43.0934) - 1.508 x_1 (x_6^2 + x_7^2) + 7.4777 (x_6^3 + x_7^3) \end{aligned}$$

##### Constraints

Several constraints ensure the mechanical integrity of the system:

$$\begin{aligned} g_1(x)= & \frac{27}{x_1 x_2^2 x_3} - 1 \le 0, \quad g_2(x) = \frac{397.5}{x_1 x_2^2 x_3^2} - 1 \le 0, \quad g_3(x) = \frac{1.93 x_4^3}{x_2 x_3 x_6^4} - 1 \le 0 \\ g_4(x)= & \frac{1.93 x_5^3}{x_2 x_3 x_7^4} - 1 \le 0, \quad g_5(x) = \frac{x_2 x_3}{40} - 1 \le 0, \quad g_6(x) = \frac{5 x_2}{x_1} - 1 \le 0 \\ g_7(x)= & \frac{x_1}{12 x_2} - 1 \le 0, \quad g_8(x) = \frac{1.5 x_6 + 1.9}{x_4} - 1 \le 0, \quad g_9(x) = \frac{1.5 x_7 + 1.9}{x_5} - 1 \le 0 \end{aligned}$$

##### Design variable bounds

The decision variables are subject to the following constraints:

$$\begin{aligned} & 2.6 \le x_1 \le 3.6, \quad 0.7 \le x_2 \le 0.8, \quad 17 \le x_3 \le 28, \\ & 7.3 \le x_4 \le 8.3, \quad 7.8 \le x_5 \le 8.3, \quad 2.9 \le x_6 \le 3.9, \quad 5.0 \le x_7 \le 5.5. \end{aligned}$$

This formulation ensures the optimization of the speed reducer weight while maintaining the required mechanical performance.

**Table 14:** Comparison of optimization results for the speed reducer design problem.

Algorithm	$X_1$	$X_2$	$X_3$	$X_4$	$X_5$	$X_6$	$X_7$	$f_{cost}$
COA	3.5482	0.7123	19.689	7.9445	7.9897	3.6608	5.3341	3721.78
PSO	3.5426	0.7341	23.7611	8.0123	7.9519	3.8882	5.5112	5598.64
GSA	3.3516	0.7518	26.2203	7.6158	8.3479	3.9144	5.2381	4376.21
WOA	3.5987	0.7358	19.8452	7.4775	7.9874	3.4014	5.2777	3113.03
RCOA	3.4975	0.7000	17.0000	7.4863	7.8054	3.7245	5.2853	3103.01

Table 14 compares the performance of various optimization algorithms for the speed reducer design problem. RCOA achieved the best cost of 3103.01, followed closely by WOA (3113.03), demonstrating their efficiency in identifying optimal design parameters. COA and GSA performed moderately, while PSO resulted in the highest cost (5598.64), indicating its lower effectiveness for this problem. The results confirm the superiority of RCOA and WOA in minimizing cost while maintaining feasible design constraints.

From the results, it is evident that RCOA and WOA provided the best design solutions in terms of minimizing cost while maintaining feasible values for all design variables. These findings reinforce the effectiveness of reindeer-inspired optimization techniques in engineering applications, particularly in complex design problems such as the speed reducer design.

## Conclusion

This paper introduces the Reindeer Cyclone Optimization Algorithm (RCOA), which combines swarm intelligence with the natural migratory behaviors of reindeer herds. RCOA introduces a flexible balance between exploration and exploitation, leveraging Lévy flight for enhanced global search and neighbor-based exploitation for local search. Compared to WOA, RCOA’s explicit separation of these phases and its novel random walk mechanism make it suitable for multimodal optimization problems and avoiding local optima. RCOA has consistently proven to be a highly competitive and robust optimization algorithm, delivering near-optimal solutions across a diverse range of complex problems. The mathematical results reflect RCOA’s strength in minimizing objective functions with minimal variance, making it a viable solution for solving real-world engineering and mathematical optimization problems. The results demonstrate that RCOA consistently delivers superior performance across different optimization problems. In the tension/compression spring design, RCOA outperformed WOA, PSO, and GSA with minimal variation in results. For the welded beam design, RCOA achieved the lowest optimum weight (1.4961) compared to WOA (1.7305) and GSA (3.5761), with fewer function evaluations than PSO. These findings highlight RCOA’s effectiveness in finding robust and accurate solutions, making it a competitive tool for real-world optimization challenges. The performance of RCOA was evaluated on the CEC’17 benchmark functions and compared with eight popular optimization algorithms. The results demonstrated that RCOA achieved superior performance in terms of convergence speed and solution accuracy, outperforming many existing methods. The convergence analysis illustrated that RCOA effectively maintains a balance between exploration and exploitation, allowing it to avoid local optima and converge to high-quality solutions efficiently. The statistical validation through the Wilcoxon Signed-Rank test confirmed the significance of RCOA’s performance improvements over traditional optimization methods. Future work will focus on enhancing RCOA by incorporating adaptive parameter control mechanisms and extending its applications to multi-objective and dynamic optimization problems. Future work will further explore adaptive control of the cyclone factor to improve the algorithm’s performance in dynamic environments.

## Data Availability

No datasets were generated or analysed during the current study.
